# Monitoring heart rhythms in adult males with X-linked ichthyosis using wearable technology: a feasibility study

**DOI:** 10.1007/s00403-025-03884-x

**Published:** 2025-02-06

**Authors:** Georgina H. Wren, Peter O’Callaghan, Abbas Zaidi, Andrew R. Thompson, Trevor Humby, William Davies

**Affiliations:** 1 School of Psychology, Cardiff University, Cardiff, UK; 2 University Hospital of Wales, Cardiff, UK; 3 South Wales Clinical Psychology Doctoral Programme, Cardiff and Vale University Health Board, Cardiff, UK; 4 School of Medicine, Cardiff University, Cardiff, UK; 5 Neuroscience and Mental Health Innovation Institute, Cardiff University, Cardiff, UK; 6 Cardiff University School of Medicine, Hadyn Ellis Building, Maindy Road, Cardiff, CF24 4HQ UK

**Keywords:** Atrial fibrillation, Premature ventricular contractions, Steroid sulfatase, Stress

## Abstract

The rare dermatological condition X-linked ichthyosis (XLI) is associated with a substantially-increased risk of cardiac arrhythmias. Arrhythmias predispose to multiple serious health conditions, and there is a need to identify them at an early stage, ideally using non-invasive, convenient, cost-effective, and reliable wearable technology methods. We tested the feasibility of monitoring heart rhythms using an Apple Watch in five adult males from the United Kingdom diagnosed with XLI. Participants returned routine electrocardiogram (ECG) traces from the watch three times per week for eight weeks (plus any traces associated with ‘watch alerts’ indicating arrhythmic episodes) for cardiologist review. Participant feedback was also obtained on the perceived usability/wearability of the watch/app, and on the study requirements. Participants were generally happy with the study conduct, found the watch/app straightforward to use, and there was a > 95% data return rate. Minor protocol improvements were suggested. There were few issues reported with wearability of the watch. Three participants exhibited no ECG abnormalities, but two presented with ventricular ectopic beats (VEs). Only one watch ‘alert’ ECG was returned, and this was considered normal sinus rhythm, suggesting the possibility of ‘false positive’ calls. Based on our small sample, smartwatch-based screening for cardiac rhythm abnormalities in the high-risk male XLI population appears useful, feasible, and acceptable to patients and a larger-scale clinical trial is warranted. Potentially, such devices may be more suited to detecting persistent abnormalities rather than unpredictable, acute, arrhythmic episodes. The high VE burden in some participants with XLI is consistent with septal defects underlying arrhythmia risk.

**Supplementary Information:**

The online version contains supplementary material available at 10.1007/s00403-025-03884-x.

## Introduction

X-linked ichthyosis (XLI) is a rare dermatological condition almost exclusively affecting males. It is characterised by abnormal desquamation and a retention hyperkeratosis, with affected individuals often presenting with large, dark brown, tightly adherent scales [8]. The biochemical mechanism underlying XLI is an accumulation of cholesterol sulfate in the stratum corneum as a consequence of deficiency for the enzyme steroid sulfatase; this deficiency typically arises due to microdeletions at Xp22.31 spanning the X-linked *STS* gene [18]. The prevalence of diagnosed XLI has been estimated at 1 in 3000–6000 males, and the prevalence of the typical microdeletion at 1 in 1500 males, implying that in many cases STS deficiency does not result in clinically-identifiable skin disease (or that skin disease is misdiagnosed) [10, 18].

XLI has been associated with a number of extracutaneous symptoms. With respect to neurological function, affected individuals demonstrate broadly equivalent IQ to that of non-affected individuals, but appear to be more commonly affected by attentional difficulties and memory deficits [18, 21]. We have recently shown that men and boys diagnosed with XLI, or carrying the associated genetic variants, are at significantly elevated risk of arrhythmias, with up to 35% of such individuals potentially affected at some point in their lives; we further identified stress as a commonly-reported precipitating factor for arrhythmic episodes [4, 20]. We estimate that Xp22.31 deletions may explain 1 in 300 cases of idiopathic atrial fibrillation (AF, the most common supraventricular arrhythmia) in middle-aged males [17].

Heart rhythm abnormalities can result in turbulent blood flow through the heart and increased risk of thrombus formation and subsequent embolism; they have been associated with vulnerability to heart failure, stroke and accelerated cognitive decline/dementia [1]. From a patient’s perspective, it is important that arrhythmias are identified and treated early to mitigate the risk of multiple long-term health conditions; treatment typically involves a combination of medication, cardioversion, and surgery [9]. Early identification and intervention are also beneficial in terms of health and social care costs. Our previous work showed that males with XLI recognise the long-term health implications of arrhythmias and the potential value of cardiac screening [20].

Arrhythmias can be associated with symptoms such as palpitations, dizziness, nausea and breathlessness, but in many cases can be asymptomatic [15, 17]. The most effective way to diagnose an arrhythmia is through electrocardiography (ECG) under baseline, stressful, or ambulatory conditions. In the gold standard monitoring paradigm, readings would be taken by a cardiologist/electrophysiologist; this can be done either non-invasively or invasively. Non-invasive monitoring is intrusive, difficult to sustain for prolonged periods, and is occasionally associated with skin rashes and discomfort. Invasive monitoring consists of surgical implantation of a continuous loop recorder which is a day-case procedure, performed under local anaesthesia. Recently, there has been a move towards using home-based or wearable technologies in patients or research study participants to monitor heart rhythms in a non-invasive, continuous, cost-effective and accessible manner [12]. These wearable technologies include smartwatches, wrist-mounted devices which record heart rate and rhythm using an opto-electrical heart sensor, and produce an ECG similar to a single-lead ECG. The Apple Watch is a Food and Drug Administration (FDA)-approved medical device and performs favourably compared to a standard 12-lead ECG; in initial trials, there is evidence that it provides a cost-effective way to discriminate reasonably effectively between sinus rhythm and atrial fibrillation [2, 6, 11, 14]. Wearable technologies, including smartwatches, may also be used to monitor dermatological phenotypes, and in the future, may monitor dermatological and cardiac measures simultaneously [3], which will clearly be of relevance to multisystem conditions like XLI.

In this study, we examined the feasibility of monitoring heart rhythms in males with XLI using the Apple Watch, with a view to ultimately running a large-scale trial to determine the utility of this mode of monitoring for early detection of arrhythmias (and particularly asymptomatic arrhythmias) within this high-risk population; given the skin condition in XLI, we were interested in whether watch-wearing would exacerbate this. We believe that a watch-based monitoring system, whilst potentially susceptible to missing certain arrhythmias or transient pathologies, could provide a quick, non-invasive, convenient and cheap mechanism for detecting arrhythmias which may be confirmed, or complemented by other, more complex approaches e.g. chest belts.

## Methods

### Aims

The key aims of the study were: (i) to assess participant engagement through response rate (particularly in light of potential cognitive issues within this population) and to gather feedback regarding frequency/timing of readings, (ii) to determine whether the device and associated application (app) can be used correctly by participants and to identify any issues associated with using the device on ichthyotic skin, (iii) To streamline procedures for delivery/return of monitoring device, for return of relevant data from participant to researchers/clinicians, and for clinician advice to participants, and (iv) to collect initial data and to identify the frequency, timing, nature, and precipitants of any arrhythmic episodes.

### Ethical approval and participants

This study was ethically-approved by Cardiff University School of Psychology Ethics Committee (EC.22.09.20.6621RA and EC.21.03.09.6302GR) and participants provided written informed consent for involvement in the research and for publication of the study findings. Recruitment and testing was conducted between December 2022 and January 2024. We recruited participants through advertising to individuals who had participated in our previous research studies, through the Ichthyosis Support Group UK, and through relevant social media patient support groups. The study inclusion criteria were as follows: adult males (aged 18-80yrs) with a confirmed clinical diagnosis of X-linked ichthyosis, possession of an iPhone Series XS (or later), willingness to download and use a new application (app), willingness to wear an Apple Watch for eight weeks, to complete a series of short online questionnaires, and to return data regarding their heart rhythm at regular intervals to the research team. There were no exclusion criteria.

### Procedure

**Fig. 1:**
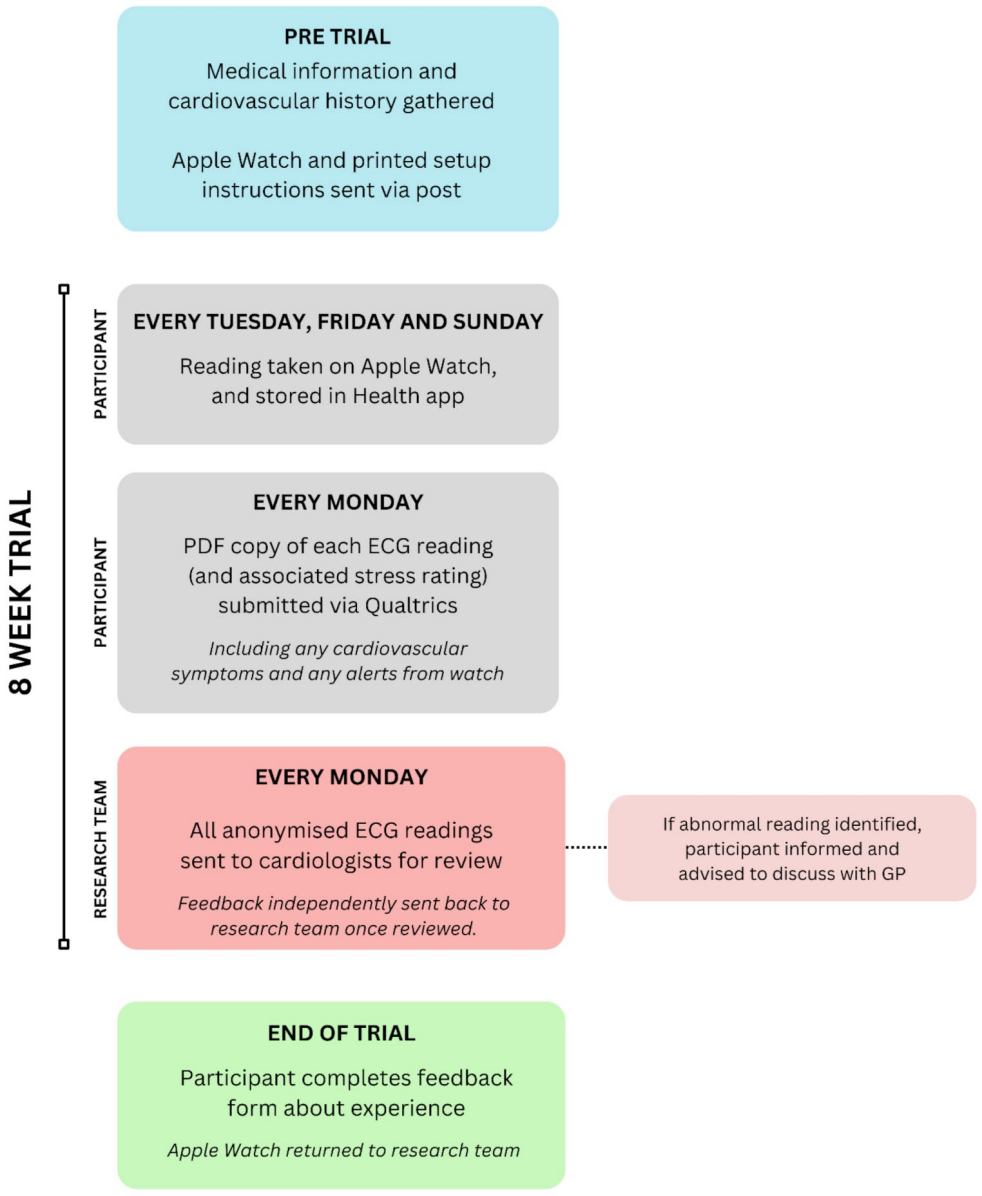
Flowchart showing participant activities and data collection timelines

The study protocol is summarised in Fig. 1. Interested participants were initially asked to complete an online screening questionnaire to confirm that they met the inclusion criteria and to acknowledge their understanding that the study might identify clinically-relevant findings but that no formal direct medical guidance would be provided. This screening questionnaire also asked participants about existing cardiac (and other medical) conditions. Participants were asked to provide their contact details for the research team.

Participants were subsequently sent an Apple Watch Series 8, charging equipment and printed instructions for setting up the watch and taking ECG readings, together with troubleshooting information. Technical details of the watch monitoring procedures are available here: https://www.apple.com/health/pdf/Heart_Rate_Calorimetry_Activity_on_Apple_Watch_November_2024.pdf. Participants were asked to wear the watch continuously for eight weeks, and to take three routine readings per week (at any time on Tuesday, Friday and Sunday). Readings were saved as a pdf file in the ‘Health’ iPhone app and were returned to the research team weekly. The research team sent out regular e-mail reminders to encourage submission. If the watch detected any irregular heart rhythm and notified the participant, this reading was automatically saved in the app, and participants were also asked to submit this reading to the research team.

For each reading submitted, participants were asked to report the time, date and circumstances of recording, and how stressed they felt (rated on a scale from 1 to 9, where 1 = not stressed at all and 9 = extremely stressed). Participants were also asked to self-report any new heart-related symptoms each week (e.g. palpitations, chest pain, breathlessness etc.)

Each week, readings were independently reviewed by two National Health Service (NHS) consultant cardiologists with particular expertise in diagnosing arrhythmias. Where findings of potential clinical relevance were identified, participants were advised to contact their doctor; however, no tailored medical advice was offered as part of this study.

Once participants had completed all eight weeks of the trial, they were asked to wipe the data stored on the Apple Watch, return it to factory settings, and return it to the research team. Finally, participants completed a short feedback form. This form initially gauged their opinion on the frequency of monitoring, ease of data return and engagement with the study (rated from 0 to 10, 0 = not very satisfied and 10 = very satisfied); it then asked about ease of watch/app usage (rated 0–10, 0 = very difficult to use, 10 = very easy to use). Three questions were then asked about comfort issues with wearing the watch (rated 0–10, 0 = strongly disagree, 10 = strongly agree). A further question then asked about participants’ happiness with returning sensitive clinical and health information to the research team (rated 0–10, 0 = not happy, 10 = very happy). Finally, in free text responses, participants were able to provide their thoughts on cardiac screening generally, on using wearable technologies to facilitate screening, and to elaborate on any of their previous answers.

Upon completion of the study, participants were awarded a £50 online shopping voucher; participants were aware of this incentive from reading the Participant Information Sheet presented before the online screening questionnaire.

### Analysis

Quantitative data are presented as median scores or mean values ± standard error of the mean.

## Results

### Recruitment and participant demographics and medical history

Seven prospective participants initially responded to our study advert. One participant (Participant 1) possessed an incompatible iPhone and could not be included in the study; a second (Participant 4) completed the screening questionnaire and was sent the watch, but did not return any ECG trace readings and was therefore withdrawn from the study. Our final sample represents > 1 in 500 individuals from the estimated target population, and, as such, compares favourably with other feasibility studies. The mean age of these five participants was 50.6 ± 7.4yrs. All participants reported a previous history of cardiovascular issues including heart rhythm abnormalities and a range of additional diagnoses (Table 1).

**Table 1:** Participants’ ages and previous cardiovascular and non-cardiovascular diagnoses

Participant number	Age (yrs)	Pre-existing cardiovascular conditions and treatments	Other pre-existing medical conditions and treatments
**2**	45	Hypertension (managed with anti-hypertensive medication)	None reported
**3**	63	Three previous episodes of atrial fibrillation (AF) in 2011 currently managed with daily anticoagulant and anti-hypertensive medication; previous experience of ‘missed beats’	Diabetes (managed with medication)
**5**	49	Three previous episodes of AF, first in 2022; latter two episodes resolved within one hour	None reported
**6**	27	Paroxysmal AF	Underactive thyroid, hayfever and depression (all managed with medication)
**7**	69	Hypertension; supraventricular ectopic beats associated with dizziness and pain (managed with calcium channel blocker and diuretic medication). Benign systolic heart murmur treated with cardiac cauterisation at age 21years	Hiatus hernia/acid reflux and glaucoma (both managed with medication); dyslexia

### Data return rates

Participants were asked to upload baseline ECG recordings three times per week for eight weeks (i.e. 24 readings in total), together with any ECG recordings associated with a watch alert. Just 5% of readings were not uploaded due to participants forgetting, or being unable, to take a reading. The majority of readings (27%) were taken in the morning (between 9am-12pm), with the remainder distributed evenly between 6am-12am. Return rate data and characteristics are summarised in Table 2.

**Table 2:** Data return rates and characteristics

Participant number	Routine recordings returned	Time of routine readings (morning: 6am-12pm, afternoon: 12pm-6pm, evening: 6pm-12am)	Mean self-reported stress level for routine readings (0–9)	Number of additional alerts	Details of alert
**2**	24/24	19 (79%) morning, 3 (13%) afternoon, 2 (8%) evening	1.1	0	-
**3**	23/24	6 (26%) morning, 6 (26%) afternoon, 11 (48%) evening	3.5	0	-
**5**	22/24	13 (59%) morning, 5 (23%) afternoon, 4 (18%) evening	1.8	0	-
**6**	22/24	8 (36%) morning, 9 (41%) afternoon, 5 (23%) evening	2.7	1	None provided; ECG trace not uploaded
**7**	24/24	7 (29%) morning, 10 (42%) afternoon, 7 (29%) evening	4.3	1	Rapid heart beat; ECG classified as normal sinus rhythm by cardiologists

### Perception and analysis of heart-related symptoms in participants

Three out of the five participants reported heart-related symptoms (‘heart palpitations, pain in chest, breathlessness etc.’) during the trial (Table 3). Participant 2 reported such symptoms in Week 2 following cold symptoms and a positive COVID-19 test. Participant 3 reported such symptoms in Weeks 1–4, but again no alerts were identified. Participant 7 reported such symptoms in Weeks 3 and 4, only the latter of which resulted in an alert on the watch. The ECG trace associated with this alert was subsequently rated as ‘normal sinus rhythm’ by both cardiologists.

**Table 3:** ECG trace interpretation

Participant number	Normal sinus rhythm in routine readings	Abnormal ECG traces in routine readings	Additional comments
**2**	All readings in Weeks 1 and 4–8 and reading 1 of Week 2 considered normal sinus rhythm by both cardiologists	Readings 2 and 3 (Week 2) considered normal sinus rhythm by one cardiologist, but to include frequent unifocal ventricular ectopic beats (VEs) by second cardiologist In Week 3, both cardiologists reported readings 1 and 2 to be characterised by frequent unifocal VEs, and reading 3 to be characterised by a single/few VEs	Abnormal traces were preceded by cold symptoms and positive Covid-19 test in Week 2
**3**	All readings in Weeks 1 and 2 considered normal sinus rhythm by both cardiologists	All readings in Weeks 3–8 considered sinus rhythm with VEs (frequent for 17/18 readings) by both cardiologists	In Weeks 1–4, participant reported breathlessness, palpitations, and a perceived irregular heartbeat. In Week 3 they were unsure whether they had received a watch alert, and in Week 4 they attended the hospital Accident and Emergency Department for an ECG and blood tests
**5**	All readings were considered by both cardiologists to be normal sinus rhythm	None	One cardiologist noted bradycardia (45-55 bpm) for 6 readings; in Week 6, one cardiologist noted some periods of sinus arrhythmia
**6**	All readings were considered by both cardiologists to be normal sinus rhythm	None	One cardiologist noted bradycardia (55 bpm) for two readings
**7**	All readings were considered by both cardiologists to be normal sinus rhythm	None	-

### Identification of abnormal ECG traces and onward referral

**Fig. 2:**
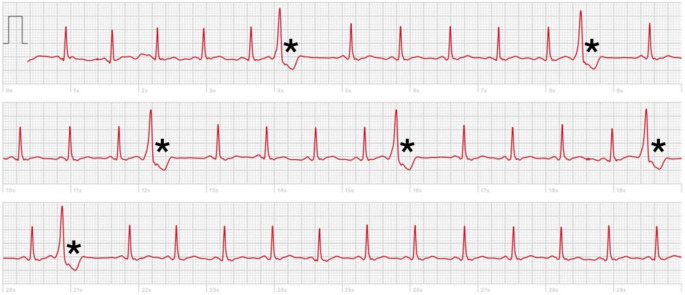
ECG trace obtained from Apple Watch for Participant 3 (Week 8, Reading 3) showing sinus rhythm with frequent ventricular ectopic beats (indicated by asterisks)

The vast majority of traces were interpretable by the cardiologists with just two showing evidence of artefact. All readings submitted by Participants 5, 6 and 7 were interpreted as ‘normal sinus rhythm’ (Table 3). Three out of 23 readings (13%) submitted by Participant 2 were regarded by both cardiologists as indicating the presence of one or more unifocal ventricular ectopic beats (VEs) (Table 3); whether these three abnormal traces were attributable to XLI and/or to a previous COVID-19 infection could not be determined. 21/26 readings (80%) submitted by Participant 3 were regarded by both cardiologists as being indicative of sinus rhythm with frequent unifocal VEs (Table 3; Fig. 2). VEs are commonly observed, particularly in older men, and often have no pathological cause; however, their persistent occurrence at high frequency can reflect underlying structural heart issues and a significantly greater risk of morbidity and mortality [16]. As such, this participant was contacted post-study, and advised to contact their General Practitioner (GP) with a view to a referral to a cardiologist. Following referral to cardiology, the individual was fitted with a Holter heart monitor for 24 h which revealed results consistent with our analysis i.e. 13% of all heartbeats recorded as VEs, and < 1% recorded as ‘supraventricular ectopic beats’. These results were returned to the individual’s GP to establish an appropriate treatment plan.

### Relationship between ECG traces and self-reported stress levels

Overall, participants self-reported low levels of stress at the point of taking readings (average < 3 out of 10), and there was no difference on this measure when comparing non-sinus rhythm versus sinus rhythm ECG traces (2.7 ± 1.8 vs. 2.0 ± 2.0).

### Participants’ perceptions of the overall trial and ease of use of the apple watch and app

Overall, participants were extremely happy with the conduct of the trial, providing median ratings of 10/10 for ‘Frequency of monitoring’, ‘Ease of data return’ and ‘Engagement with study’. We note the possibility that participants’ awareness of financial compensation might have influenced their willingness to participate, or biased their feedback more favourably. Suggestions for minor improvements to the process included: the provision of a watch strap with a greater size range, greater clarity with respect to downloading and submitting ECG trace files within the study guide, clearer initial guidance regarding the make/model of phone and associated software required to participate, and more intuitive presentation of the stress level rating scales. Participants provided median usability ratings of 10/10 for ‘Understanding the information provided’, ‘Opening and using the ECG App’, ‘Understanding any alerts or information via the watch’ and ‘Downloading ECG outputs’. One participant commented on the short battery life of the watch.

### Wearability issues with using the watch

Participants reported that they found the watch comfortable to wear on a daily basis (median rating 10/10), and disagreed with the statements that wearing the watch made their skin condition worse or irritated their skin (median rating 1/10). Generally, participants disagreed that they found the sensation of wearing the watch every day overwhelming (median rating 1/10, although one participant did report agreement with this sentiment (score of 7/10).

### Provision of clinical/health data to the research team

Participants were happy to provide sensitive clinical/health data to the research team in the context of a study with appropriate ethical approval (median rating 10/10).

### Qualitative responses

Participants’ comments on the use of wearable technology in general, and on this study in particular, are summarised in Table 4.

**Table 4:** Qualitative feedback from participants

Aspect of study	Participant comments
View on wearable technologies	“…*from my experiences hospital staff and some GPs are not yet open to this technology*, *maybe within the next decade”* (Participant 3) *“I think it’s a fantastic idea… if the tech is reliable*, *I think it should be encouraged and could help alleviate pressure on services”* (Participant 6)
General conduct of current study	*“Very easy*, *seamless and professional”* (Participant 6) *“I found the monitoring easy and I was pleased to join your study”* (Participant 7)
Monitoring and data return procedures	*“I would like some feedback from yourselves*, *as we are not experts in reading ECGs”* (Participant 3) *“Although taking ECGs three times a week was manageable*, *I don’t know whether monitoring for a set time period would provide more accurate data”* (Participant 5) *“I think wearing ECG monitor might work better. I wore a Holter Monitor that picked up discrepancies in my heartbeat”* and *“I was never notified by the watch or iPhone even though I could feel them [irregular beats]”* (Participant 7)

## Discussion

Genetic deletions at Xp22.31 associated with the rare dermatological condition X-linked ichthyosis (XLI) predispose to cardiac arrhythmias. Here, we tested the feasibility of heart rhythm monitoring using an Apple Watch and associated app in a small sample of adult males with a clinical diagnosis of XLI.

The five participants we recruited all had a history of cardiovascular issues (arrhythmia or hypertension). One participant presented with acid reflux/hiatus hernia and one with hayfever (allergic rhinitis), somewhat consistent with our previous observation that individuals carrying XLI-associated deletions and exhibiting heart rhythm abnormalities tend to present disproportionately with gastrointestinal and atopic comorbidities [20]. Theoretically, variability in participant characteristics (e.g. with respect to age or medical conditions) could complicate the interpretation, and generalisability, of the current findings.

Our first main finding was that participants were highly-engaged in the research area and this particular study, and complied impressively with the requirements of the latter; they demonstrated an ability to use the watch and associated app, and there appeared to be few adverse effects of wearing the watch on skin health. As such, compliance, technological, and cognitive issues within the XLI population are unlikely to impact adversely upon the conduct of future studies with similar demands, particularly if a more comprehensive ‘set-up guide’ is provided. One participant reported that he found the sensation of wearing the watch continuously somewhat overwhelming. In future work, participants might be advised that if continuous watch-wearing becomes distressing, the device should only be worn to take readings. This consideration might be particularly relevant given that autism-related traits, which can include sensory hypersensitivities, are elevated in individuals with XLI [5].

The existing study procedures for the initial recruitment of participants, despatch/return of the watch, dissemination/return of the data between participants, the research team and clinicians worked effectively. The need to recruit larger, more highly-powered, samples in future work (including international participants), would require minor amendments with respect to data privacy considerations, device shipping/return processes, and clinician investment. To reduce the burden upon cardiologists, future work might use sophisticated computer algorithms to diagnose straightforward rhythms [7], with only suspected abnormal readings forwarded for detailed review. In general, participant responses regarding the study were highly consistent; hence, for many of the measures obtained here, a larger sample size would not necessarily yield significantly more useful information.

The ECG traces collected from participants could be interpreted reliably and consistently by cardiologists. On at least one occasion, despite physical symptoms associated with an abnormal heart rhythm being present, no watch alert was received; conversely, the one alert which was received and characterised indicated normal sinus rhythm. Hence, the utility of the alert feature for detecting genuine unexpected arrhythmic episodes in individuals with XLI remains unclear, with the possibility of both false negatives (i.e. no alert in the presence of genuine rhythm abnormalities) and false positives (i.e. watch alert in the presence of no underlying rhythm abnormalities). Single-lead ECG devices are less sensitive to some arrhythmias than multi-lead devices, and accurate arrhythmia detection may be further dependent upon user factors including skin contact, arm movement, and background ‘normal’ heart trace. However, routine monitoring using the watch did identify the continued presence of ventricular ectopic beats in two participants; these were confirmed in one participant using a more established method of clinical monitoring. As such, continuous cardiac monitoring using the Apple watch might aid in the early identification of persistent rhythmic abnormalities and facilitate early referral in individuals with XLI. We have recently proposed, on the basis of new basic science findings, clinical case reports, and anecdotal evidence from participants in social media patient support groups, that the increased risk of arrhythmias within the XLI population might be mediated via maldevelopment of the cardiac septum [19]. Interestingly, in the context of the current findings, unusually high frequencies of VEs (or premature ventricular contractions, PVCs) have been described in > 50% of middle-aged individuals with repaired or unrepaired ventricular septal defects [13]. Hence, heart rhythm disturbances more generally, and not just atrial fibrillation/flutter, might be associated with XLI. Our previous work indicated psychosocial stress as the most frequently reported trigger for arrhythmic episodes within the XLI population. Here, we found no evidence that higher self-reported stress levels were associated with abnormal ECG traces, although this analysis is clearly limited by power issues and by the low frequency of arrhythmic episodes in most participants. It is also possible that, in XLI, stress levels are more strongly-associated with acute, transient arrhythmic episodes than with ongoing, more persistent arrhythmias.

## Conclusions

This study has provided evidence that adult males with XLI are receptive to the idea of cardiac screening using wearable technologies, are competent with using such technologies, and are willing to collect and return associated data regularly over a prolonged period. Importantly, use of the watch did not appear to cause significant exacerbation of skin issues. Although the watch could be used to detect some ongoing cardiac issues, the specificity and sensitivity with which it detects all arrhythmic episodes within the XLI population remains to be determined. Participants consented to, and were happy with, the current data security arrangements (use of Health app, and transmission of data via a secure Qualtrics platform only accessible by specific members of the research team), but the nature of these arrangements might require revision for subsequent larger-scale international studies where additional, more sophisticated, data from the app might be collected (e.g. using the HealthKit API). Such follow-up work might recruit both adults and boys affected by XLI from dermatology clinics in addition to the routes employed here, might use a later version of the Apple Watch with enhanced sensitivity and specificity for arrhythmia detection, and include individuals with defined arrhythmias but without XLI as ‘positive control’ subjects.

## Electronic supplementary material

Below is the link to the electronic supplementary material.

Supplementary Material 1

## Data Availability

Data is provided within the manuscript or Supplementary Information files.
